# Haemothorax due to intercostal artery injury after thoracentesis

**DOI:** 10.1002/rcr2.950

**Published:** 2022-04-15

**Authors:** Chien‐Hong Chou, Hong‐Jen Hsieh

**Affiliations:** ^1^ Department of Internal Medicine National Taiwan University Hospital Yun‐Lin Branch Douliu Taiwan; ^2^ Department of Radiology National Taiwan University Hospital Yun‐Lin Branch Douliu Taiwan

**Keywords:** computed tomography, haemothorax, thoracentesis

## Abstract

Haemothorax is a rare complication after thoracentesis. The diagnosis of haemothorax should be considered if there is deterioration in vital signs post‐procedure. Contrast‐enhanced computed tomography is a useful diagnostic tool for bleeding source location. Transarterial embolization is a potential method to control bleeding.

We present a rare case of haemothorax complication post thoracentesis. Contrast computed tomography was used to locate the bleeding site, and transarterial embolization was performed to stop intercostal artery bleeding.

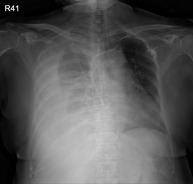

## CLINICAL IMAGE

**FIGURE 1:**
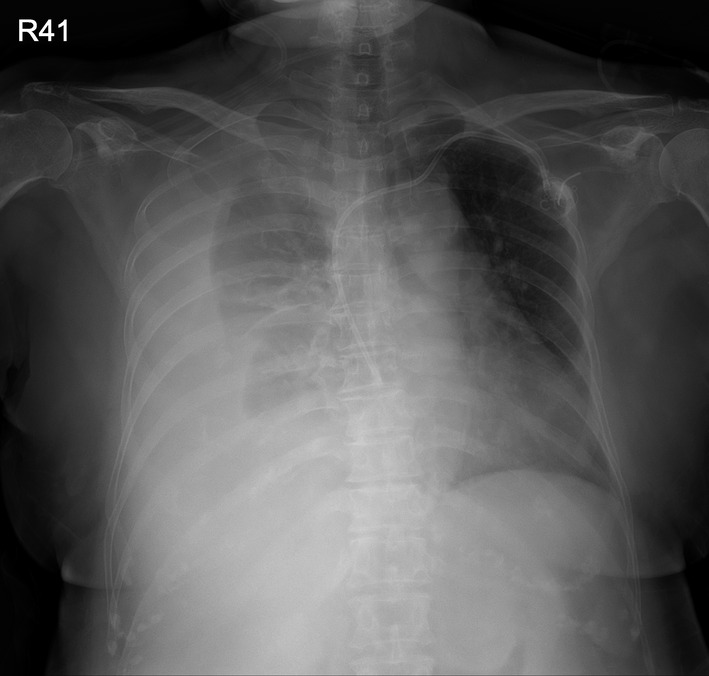
Chest roentgenogram showed right‐sided massive pleural effusion after thoracentesis

**FIGURE 2:**
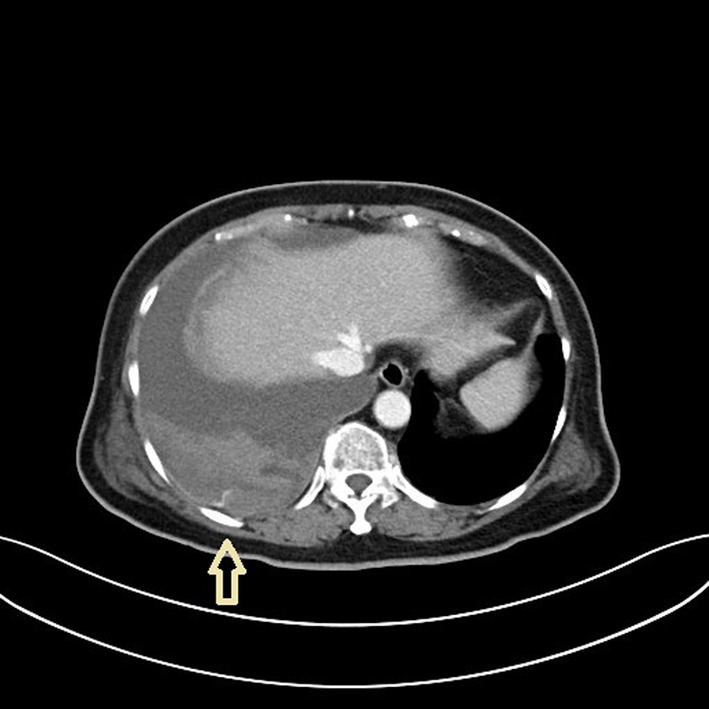
Computed tomography showed right‐sided pleural effusion and contrast medium extravasations from the right intercostal artery (arrow)

**FIGURE 3:**
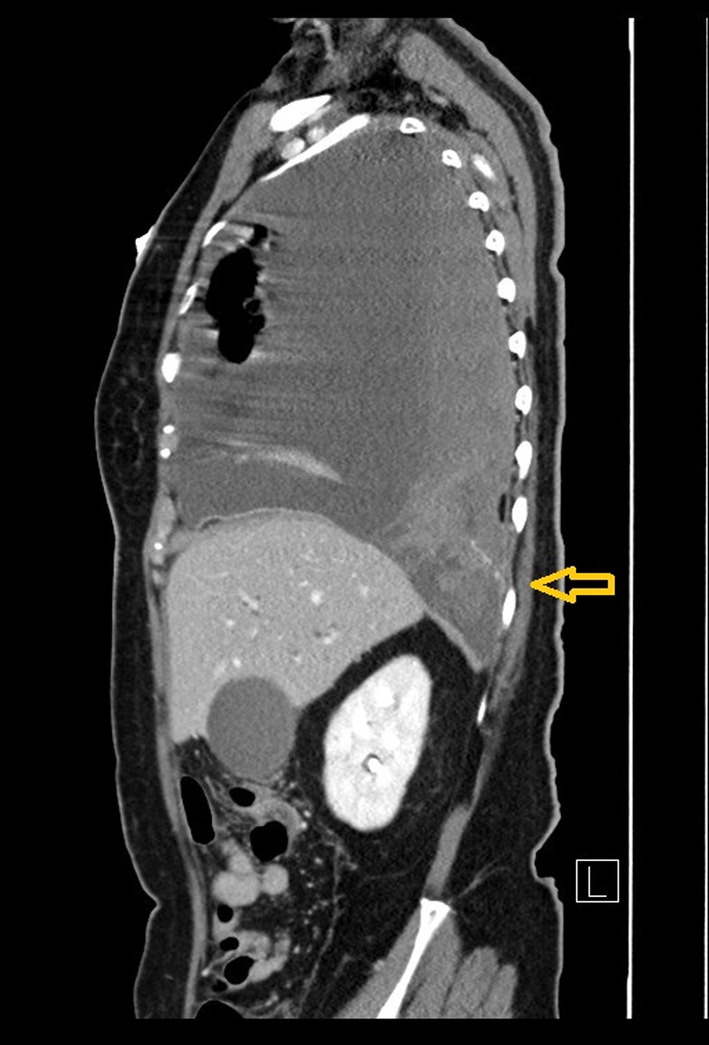
Sagittal view of computed tomography showed extravasations of contrast medium from the right 10th intercostal artery (arrow)

A 70‐year‐old woman with metastatic lung cancer and right‐sided malignant pleural effusion was hospitalized for abdominal pain. She also had a history of deep venous thrombosis and had been on anti‐coagulant medication, edoxaban, for 1 year. Computed tomography (CT) abdomen showed intra‐abdominal mass, leading to a suspicion of lung cancer metastasis. Chest roentgenogram (CXR) showed increased right‐sided pleural effusion (around 500–1000 cc). She received thoracentesis with real‐time, sonography guidance via 18‐gauge needle catheter. However, dyspnoea developed 1 day after the procedure, with arterial oxygen desaturation, hypotension and haemoglobin drop from 7.5 to 5.9 g/dl. CXR (Figure 1) revealed newly developed right‐sided massive pleural effusion. CT chest showed contrast extravasations from the right 10th intercostal artery (Figures 2 and 3), with presumed intercostal artery injury due to thoracentesis. Tube thoracostomy was performed and haemothorax drained. Transarterial embolization (TAE) was performed to stop intercostal artery bleeding. After TAE, haemothorax decreased and haemodynamic status stabilized. Haemothorax is a rare complication of thoracentesis.^1^ The risk of developing haemothorax is higher when certain conditions are present: lung infections, malignancies, coagulation disorders or anticoagulation use, such as edoxaban in our patient. Sonography‐guided thoracentesis can decrease the complication rate. New‐onset dyspnoea and haemodynamic deterioration after thoracentesis should raise the possibility of haemothorax. Early recognition and management of haemothorax decrease morbidity and mortality. Contrast‐enhanced CT is a useful tool for locating bleeding site. Management of haemothorax includes thoracentesis, tube thoracostomy or video‐assisted thoracic surgery. TAE is a potential method to stop bleeding other than traditional surgical intervention.

## CONFLICT OF INTEREST

None declared.

## AUTHOR CONTRIBUTION

Chien‐Hong Chou was responsible for conceptualization and drafting the manuscript. Hong‐Jen Hsieh provided clinical and radiological data.

## ETHICS STATEMENT

The authors declare that appropriate written informed consent was obtained for the publication of this manuscript and accompanying images.

## Data Availability

The data that support the findings of this study are available from the corresponding author upon reasonable request.
